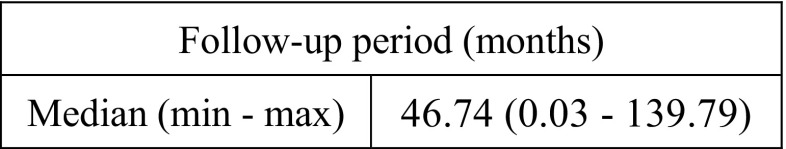# Comprehensive registry of esophageal cancer in Japan, 2012

**DOI:** 10.1007/s10388-019-00674-z

**Published:** 2019-05-16

**Authors:** Yuji Tachimori, Soji Ozawa, Hodaka Numasaki, Ryu Ishihara, Hisahiro Matsubara, Kei Muro, Tsuneo Oyama, Yasushi Toh, Harushi Udagawa, Takashi Uno, The Registration Committee for Esophageal Cancer of the Japan Esophageal Society

**Affiliations:** 1 Cancer Care Center, Kawasaki Saiwai Hospital, 31-27 Omiya-cho, Saiwai-ku, Kawasaki, Kanagawa 212-0014 Japan; 2 Department of Gastroenterological Surgery, Tokai University School of Medicine, Isehara, Japan; 3 Department of Medical Physics and Engineering, Osaka University Graduate School of Medicine, Osaka, Japan; 4 Department of Gastrointestinal Oncology, Osaka International Cancer Institute, Osaka, Japan; 5 Department of Frontier Surgery, Graduate School of Medicine, Chiba University, Chiba, Japan; 6 Department of Clinical Oncology, Aichi Cancer Center Hospital, Aichi, Japan; 7 Department of Gastroenterology, Saku General Hospital, Nagano, Japan; 8 Department of Gastroenterological Surgery, National Kyushu Cancer Center, Fukuoka, Japan; 9 Toranomon Hospital Kajigaya, Kawasaki, Japan; 10 Department of Diagnostic Radiology and Radiation Oncology, Graduate School of Medicine, Chiba University, Chiba, Japan

## Preface 2012

We deeply appreciate the great contributions of many physicians in the registry of esophageal cancer cases. The Comprehensive Registry of Esophageal Cancer in Japan, 2012, was published here, despite some delay. The registry complies with the Act for the Protection of Personal Information. The encryption with a HASH function is used for anonymity in an unlinkable fashion.

We briefly summarized the Comprehensive Registry of Esophageal Cancer in Japan, 2012. Japanese Classification of Esophageal Cancer 10th [1] and UICC TNM Classification 7th [2] were used for cancer staging according to the subjected year. A total of 8003 cases were registered from 316 institutions in Japan. Tumor locations were cervical: 4.6%, upper thoracic: 12.8%, middle thoracic: 47.9%, lower thoracic: 26.1% and EG junction: 7.6%. Superficial carcinomas (Tis, T1a, T1b) were 38.2%. As for the histologic type of biopsy specimens, squamous cell carcinoma and adenocarcinoma accounted for 89.5% and 6.0%, respectively. Regarding clinical results, the 5-year survival rates of patients treated using endoscopic resection, concurrent chemoradiotherapy, radiotherapy alone, or esophagectomy were 84.4, 32.4, 24.9, and 55.6%, respectively. The endoscopic submucosal dissection accounted for 84.9% of endoscopic resection. Esophagectomy was performed in 4722 cases. Concerning the approach used for esophagectomy, 36.0% of the cases were treated thoracoscopically. The operative mortality (within 30 days after surgery) was 0.52% and the hospital mortality was 2.35%. The 5-year survival rate of patients with pStage IV in UICC classification (including patients with supraclavicular node metastasis) was better than that of patients with pStage IVb in JES classification (not including patients with supraclavicular node metastasis).

We hope that this Comprehensive Registry of Esophageal Cancer in Japan for 2012 will help to improve all aspects of the diagnosis and treatment of esophageal cancer in Japan.

## Contents

**Fig. 1:**
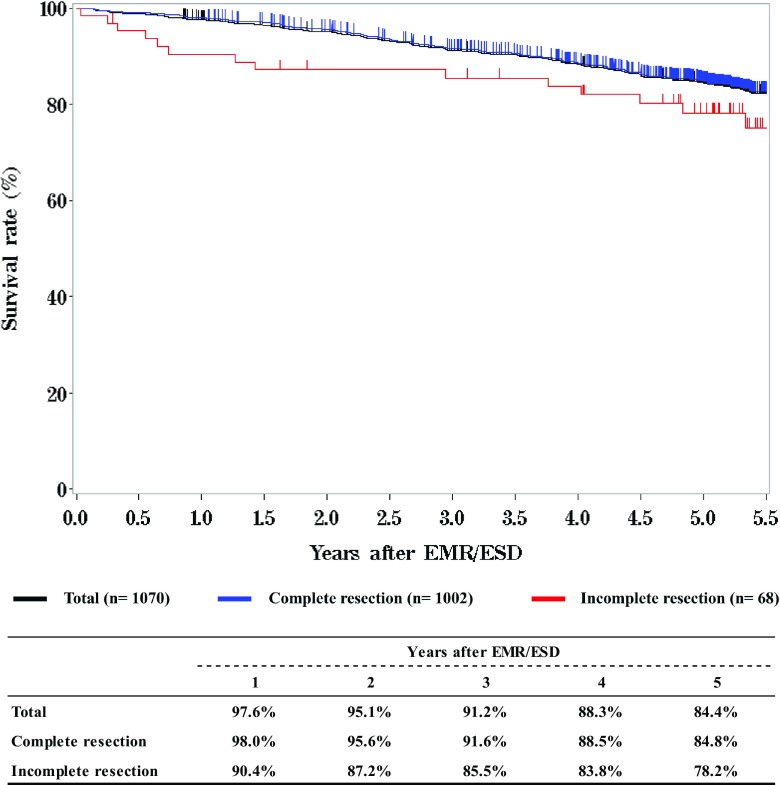
Survival of patients treated with EMR/ESD

**Fig. 2:**
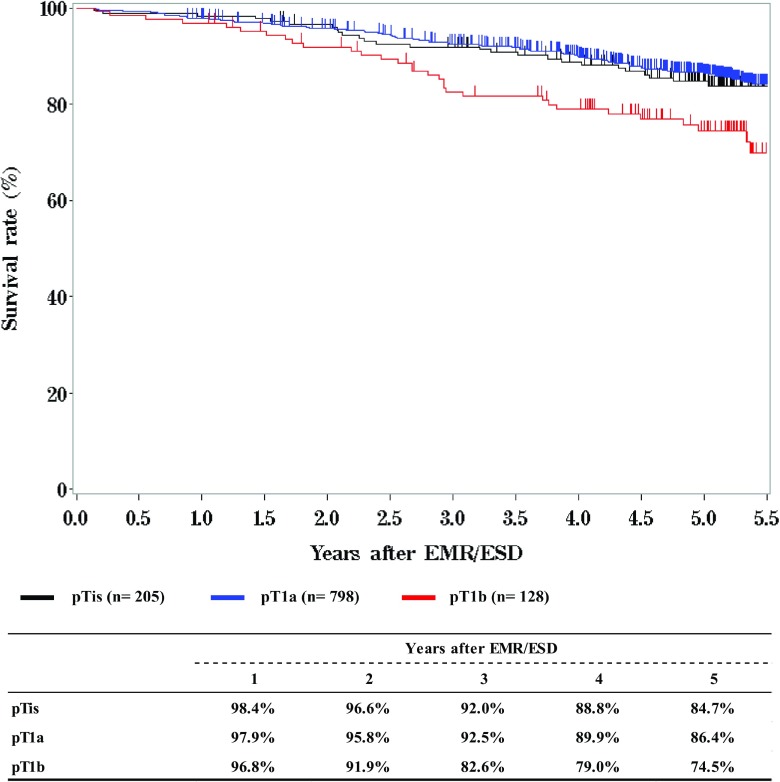
Survival of patients treated with EMR/ESD according to the pathological depth of tumor invasion, pT (UICC TNM 7th)

**Fig. 3:**
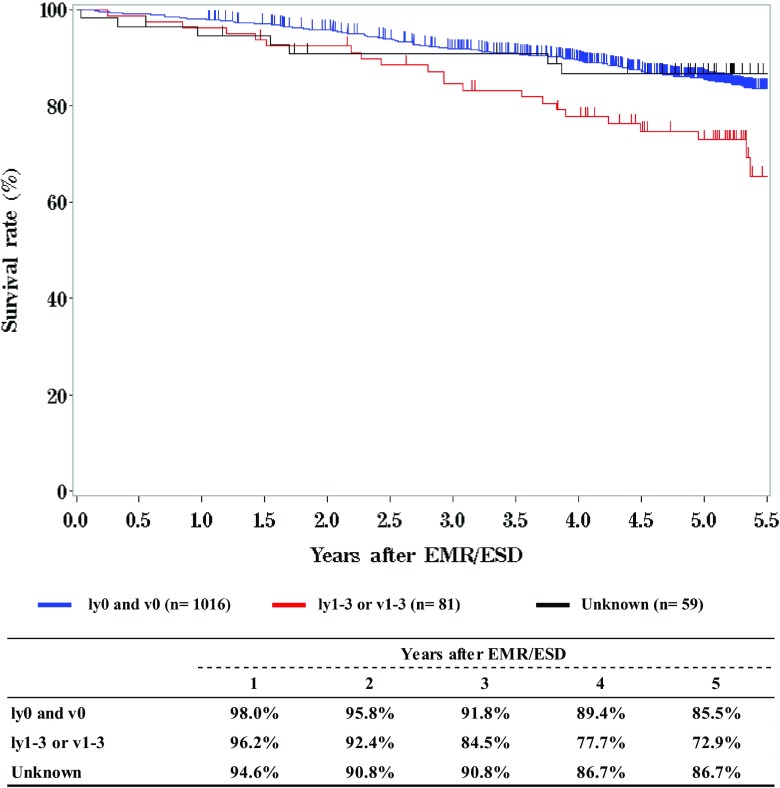
Survival of patients treated with EMR/ESD according to the lymphatic and venous invasion

**Fig. 4:**
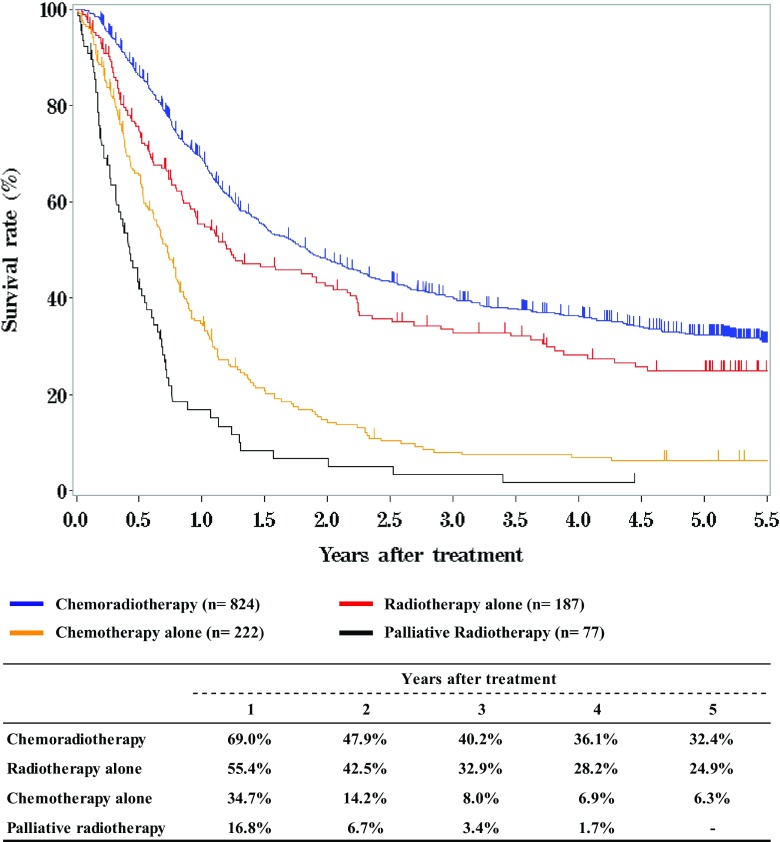
Survival of patients treated with chemotherapy and/or radiotherapy

**Fig. 5:**
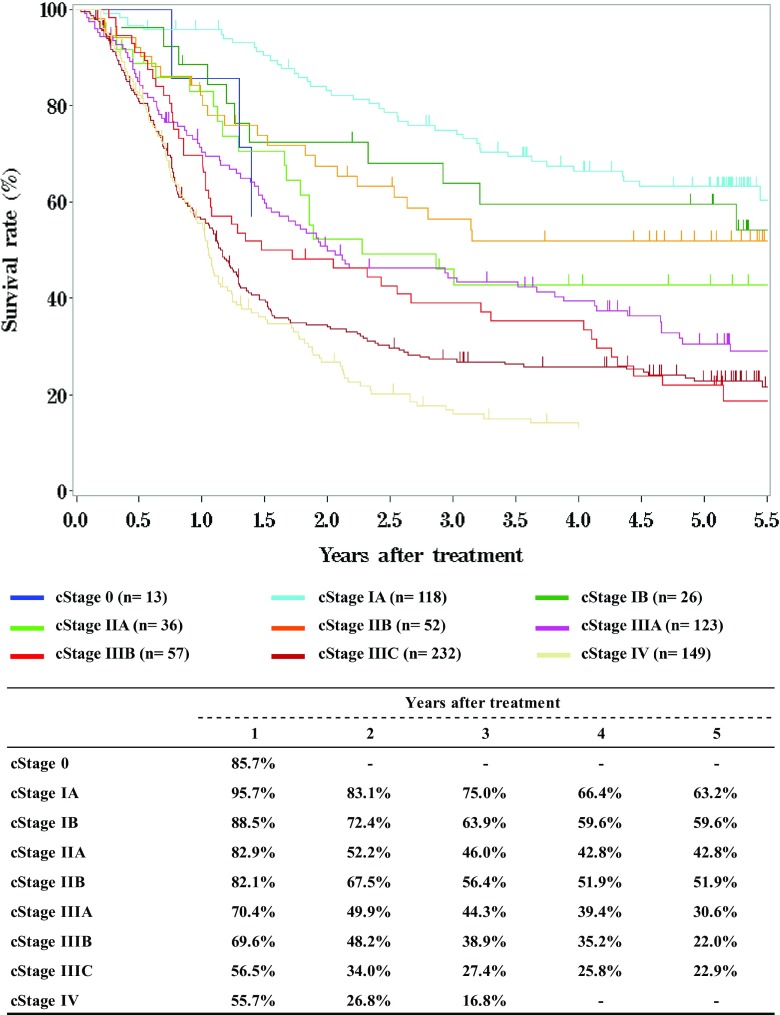
Survival of patients treated with definitive chemoradiotherapy according to clinical stage (UICC TNM 7th)

**Fig. 6:**
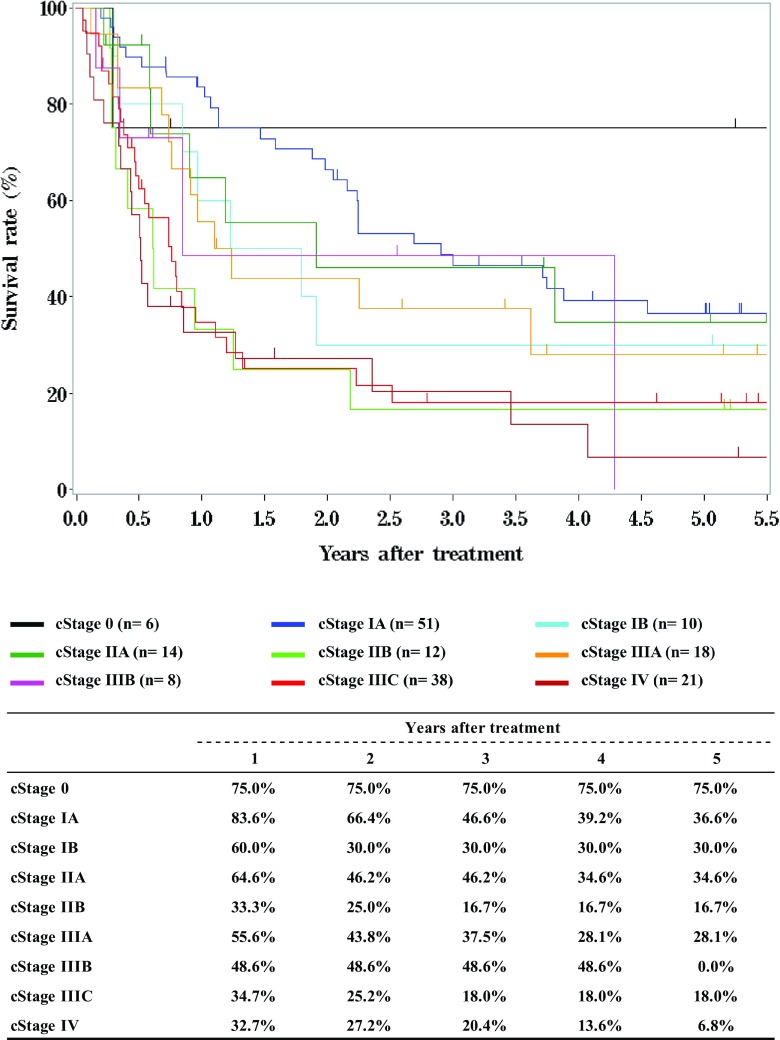
Survival of patients treated with radiotherapy alone according to clinical stage (UICC TNM 7th)

**Fig. 7:**
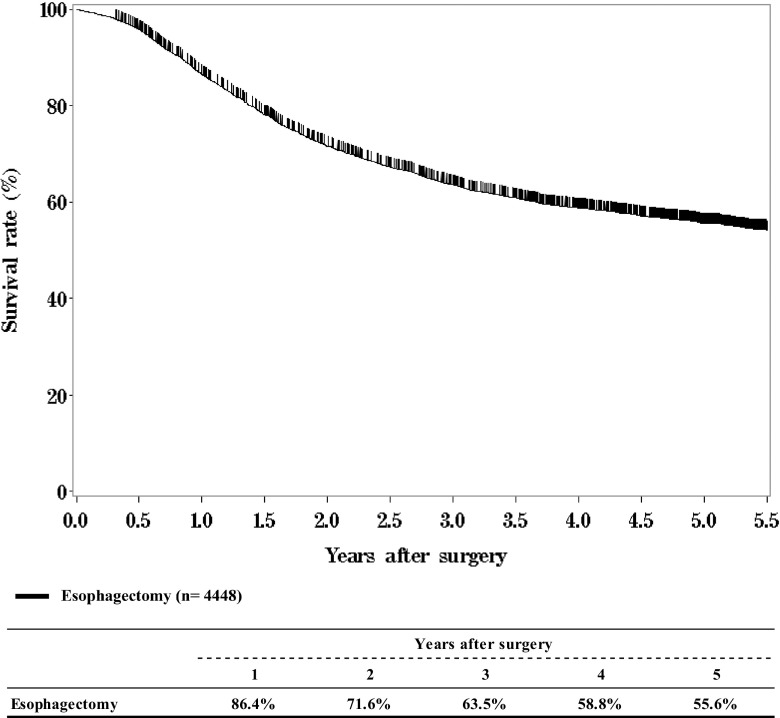
Survival of patients who underwent esophagectomy

**Fig. 8:**
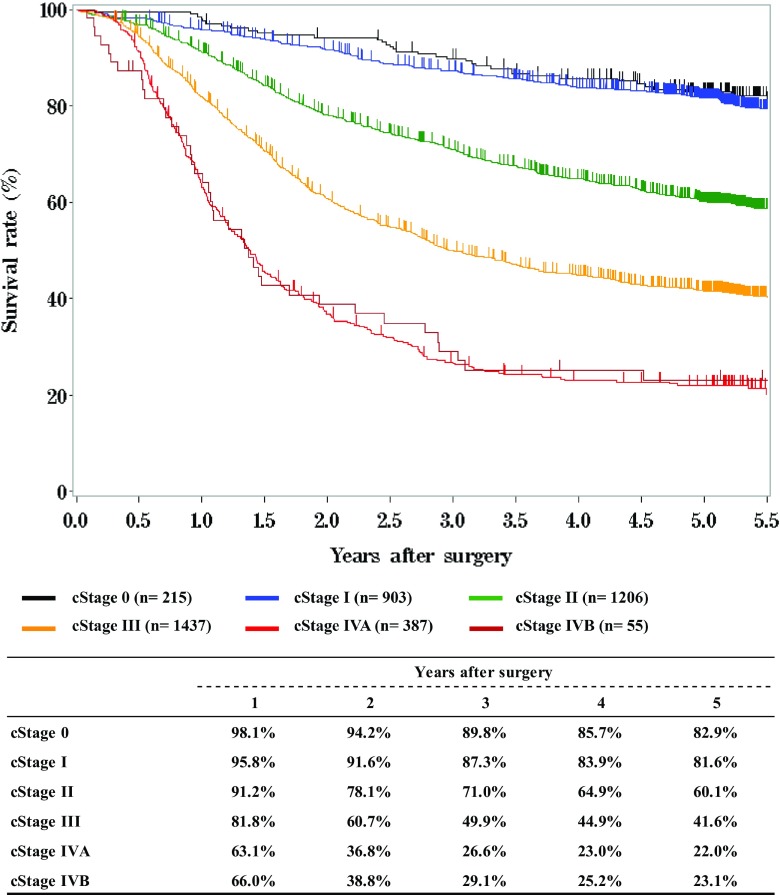
Survival of patients who underwent esophagectomy according to clinical stage (JES 10th)

**Fig. 9:**
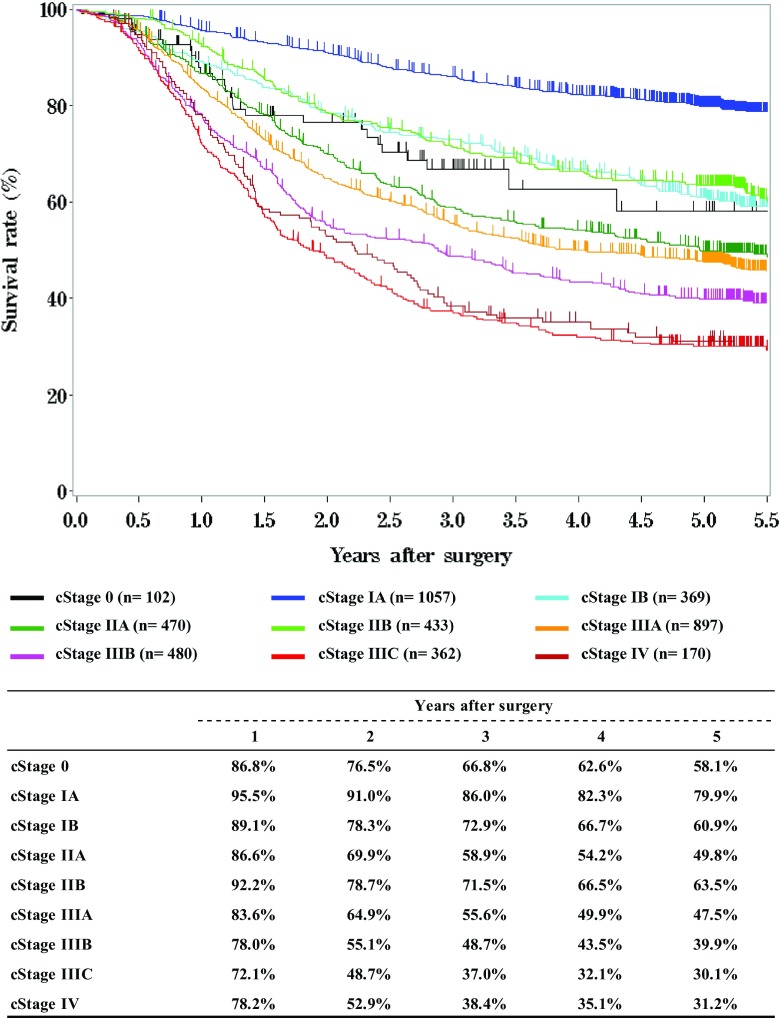
Survival of patients who underwent esophagectomy according to clinical stage (UICC 7th)

**Fig. 10:**
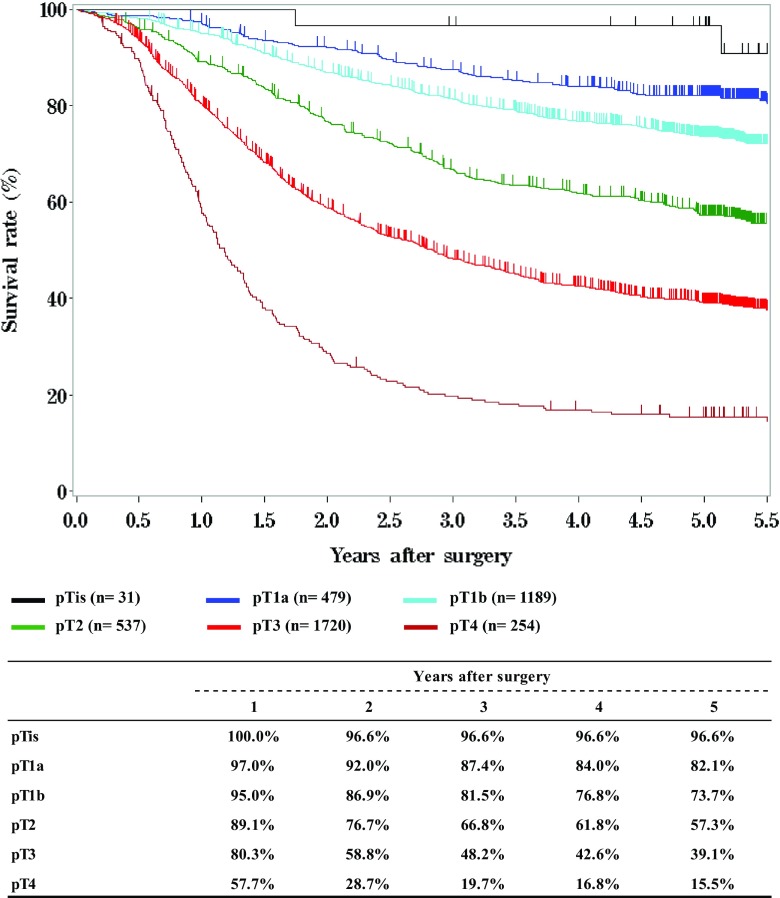
Survival of patients who underwent esophagectomy according to the depth of tumor invasion, pT (JES 10th)

**Fig. 11:**
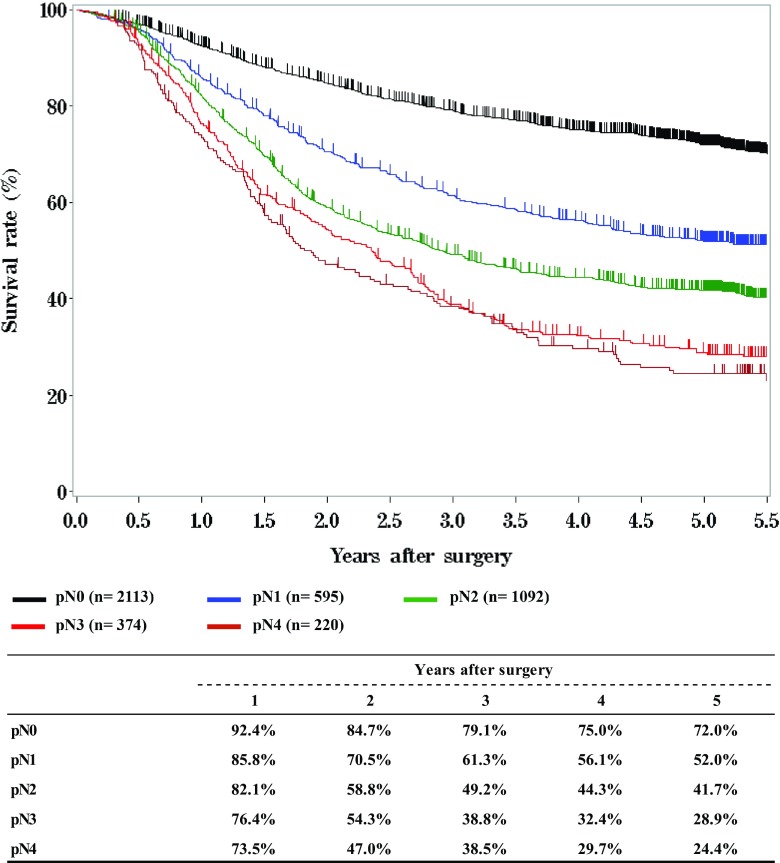
Survival of patients who underwent esophagectomy according to lymph node metastasis, pN (JES 10th)

**Fig. 12:**
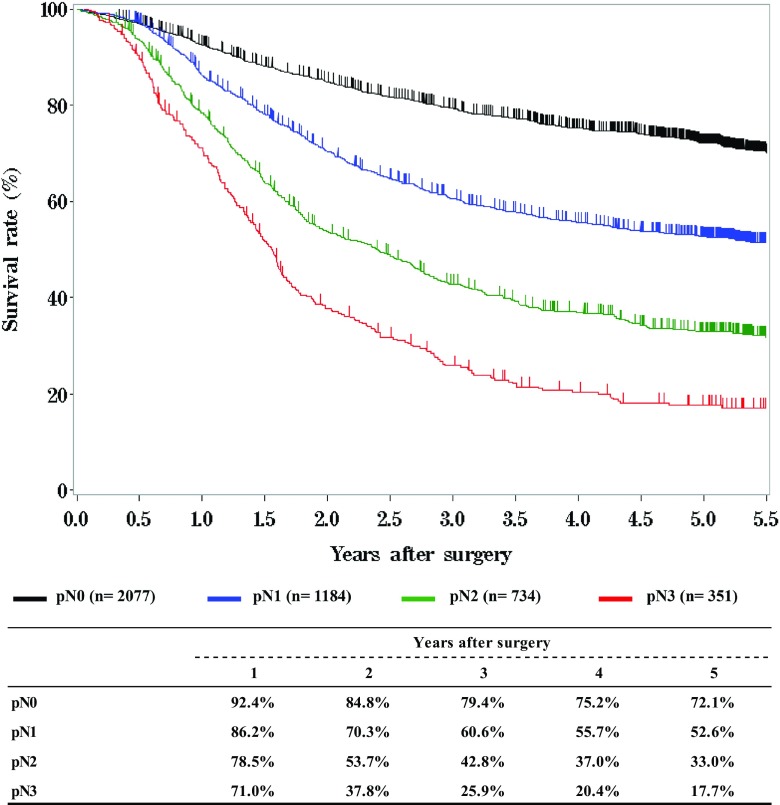
Survival of patients who underwent esophagectomy according to lymph node metastasis, pN (UICC 7th)

**Fig. 13:**
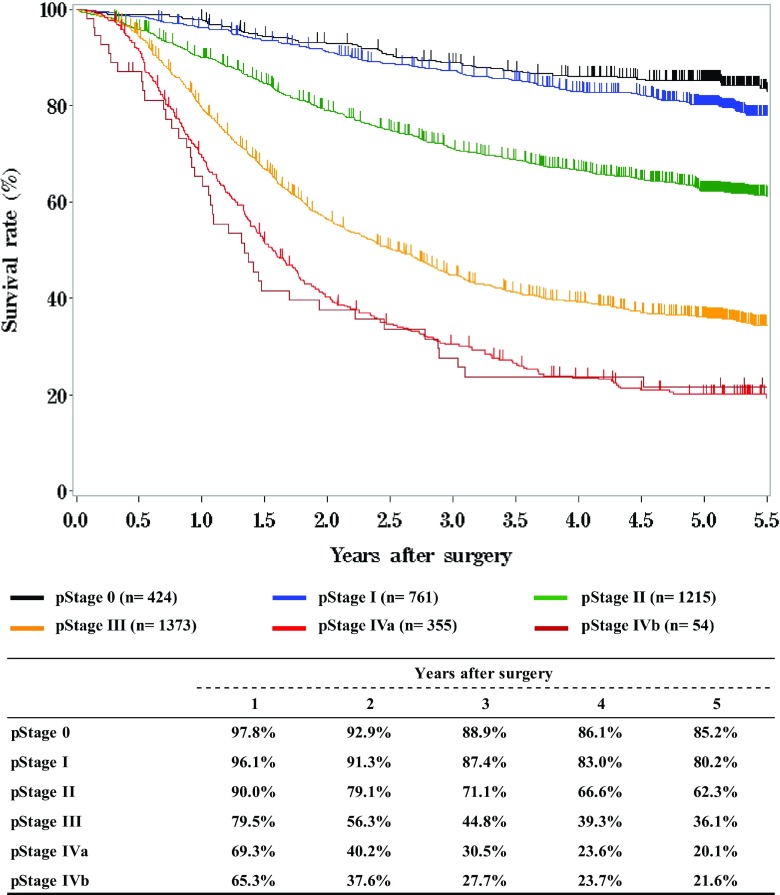
Survival of patients who underwent esophagectomy according to pathological stage (JES 10th)

**Fig. 14:**
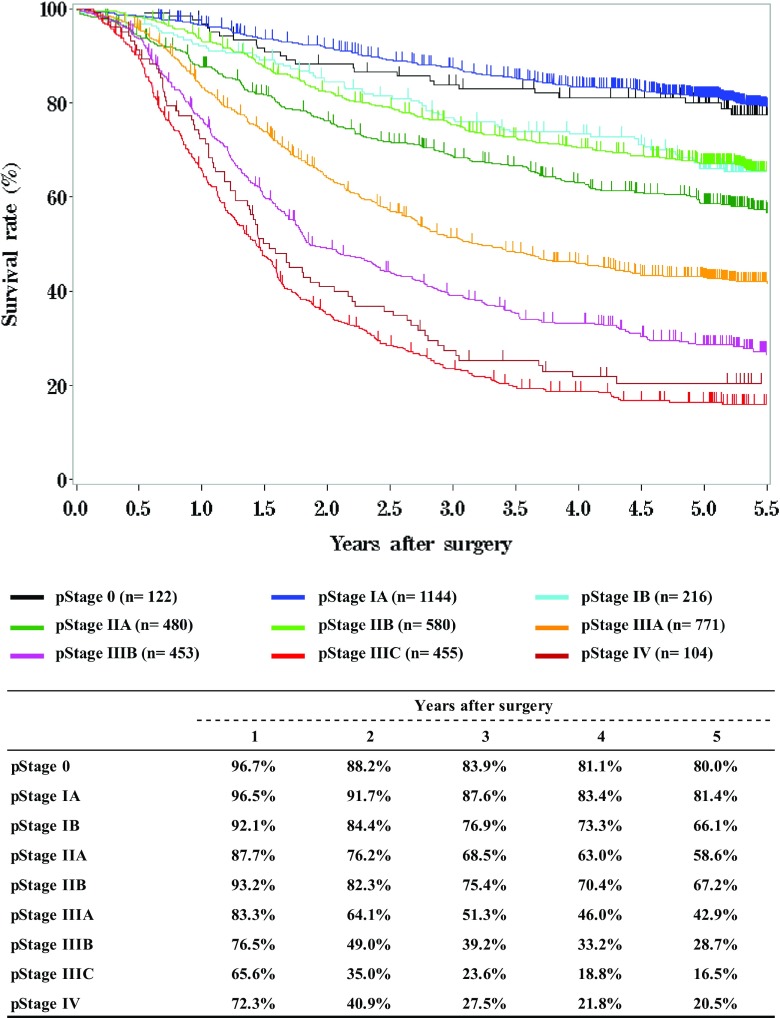
Survival of patients who underwent esophagectomy according to pathological stage (UICC TNM 7th)

**Fig. 15:**
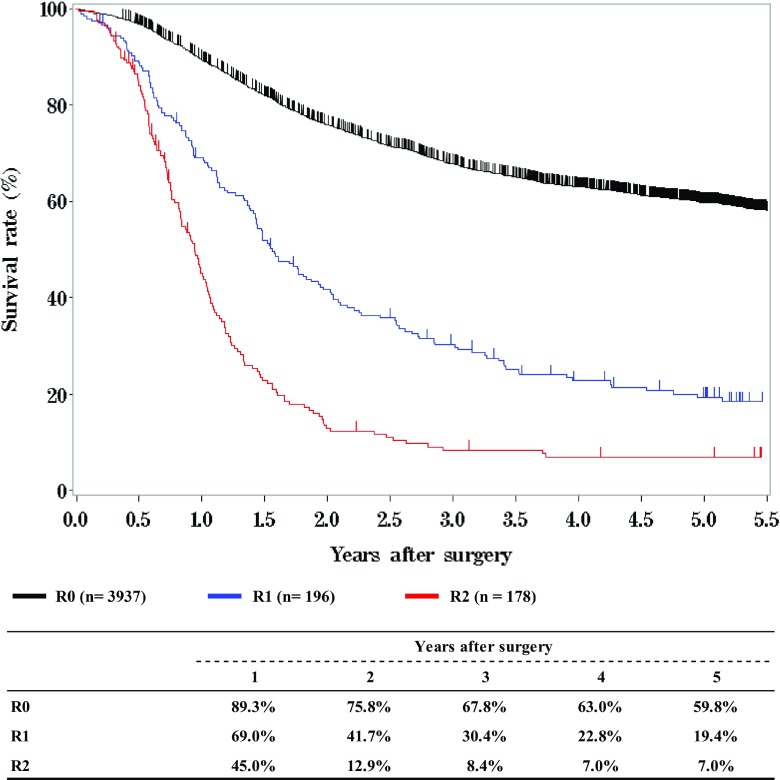
Survival of patients who underwent esophagectomy according to residual tumor (R)

I. **Clinical factors of esophageal cancer patients treated in 2012**
  1. **Institution-registered cases in 2012**
  2. **Patient background**
**Table** **1** **Age and gender**
**Table** **2** **Primary treatment**
**Table** **3** **Tumor location**
**Table** **4** **Histologic types of biopsy specimens**
**Table** **5** **Depth of tumor invasion, cT (UICC TNM 7th)**
**Table** **6** **Lymph node metastasis, cN (UICC TNM 7th)**
**Table** **7** **Distant metastasis, cM (UICC TNM 7th)**
**Table** **8** **Clinical stage (UICC TNM 7th)**
II. **Results of endoscopically treated patients in 2012**
**Table** **9** **Details of endoscopic treatment for curative intent**
**Table** **10** **Complications of EMR/ESD**
**Table** **11** **Pathological depth of tumor invasion of EMR/ESD specimens**
**Figure** **1** **Survival of patients treated with EMR/ESD**
**Figure** **2** **Survival of patients treated with EMR/ESD according to the pathological depth of tumor invasion, pT (UICC TNM 7th)**
**Figure** **3** **Survival of patients treated with EMR/ESD according to the lymphatic and venous invasion**
III. **Results in patients treated with chemotherapy and/or radiotherapy in 2012**
**Table** **12** **Dose of irradiation (non-surgically treated cases)**
**Table** **13** **Dose of irradiation (surgically treated cases)**
**Figure** **4** **Survival of patients treated with chemotherapy and/or radiotherapy**
**Figure** **5** **Survival of patients treated with definitive chemoradiotherapy according to clinical stage (UICC TNM 7th)**
**Figure** **6** **Survival of patients underwent radiotherapy alone according to clinical stage (UICC TNM 7th)**
IV. **Results in patients who underwent esophagectomy in 2012**
**Table** **14** **Treatment modalities of esophagectomy**
**Table** **15** **Tumor location**
**Table** **16** **Approaches to tumor resection**
**Table** **17** **Video-assisted surgery**
**Table** **18** **Fields of lymph node dissection according to the location of the tumor**
**Table** **19** **Reconstruction route**
**Table** **20** **Organs used for reconstruction**
**Table** **21** **Histological classification**
**Table** **22** **Depth of tumor invasion, pT (JES 10th)**
**Table** **23** **Pathological grading of lymph node metastasis, pN (JES 10th)**
**Table** **24** **Pathological findings of lymph node metastasis, pN (UICC 7th)**
**Table** **25** **Pathological findings of distant organ metastasis, pM (JES 10th)**
**Table** **26** **Residual tumor**
**Table** **27** **Causes of death**
**Figure** **7** **Survival of patients who underwent esophagectomy**
**Figure** **8** **Survival of patients who underwent esophagectomy according to clinical stage (JES 10th)**
**Figure** **9** **Survival of patients who underwent esophagectomy according to clinical stage (UICC 7th)**
**Figure** **10** **Survival of patients who underwent esophagectomy according to the depth of tumor invasion, pT (JES 10th)**
**Figure** **11** **Survival of patients who underwent esophagectomy according to lymph node metastasis, pN (JES 10th)**
**Figure** **12** **Survival of patients who underwent esophagectomy according to lymph node metastasis, pN (UICC 7th)**
**Figure** **13** **Survival of patients who underwent esophagectomy according to pathological stage (JES 10th)**
**Figure** **14** **Survival of patients who underwent esophagectomy according to pathological stage (UICC TNM 7th)**
**Figure** **15** **Survival of patients who underwent esophagectomy according to residual tumor (R)**

## I. Clinical factors of esophageal cancer patients treated in 2012

Institution-registered cases in 2012

**Table:** 

Institution
Ageo Central General Hospital
Aichi Cancer Center
Aichi Medical University Hospital
Aizawa Hospital
Akaishi Hospital
Akita Kouseiren Hiraga Hospital
Akita University Hospital
Aomori City Hospital
Arao Municipal Hospital
Asahikawa Medical College Hospital
Cancer Institute Hospital of JFCR
Chiba Cancer Center
Chiba Medical Center
Chiba University Hospital
Chibaken Saiseikai Narashino Hospital
Chigasaki Municipal Hospital
Dokkyo Medical University Hospital
Dokkyo Medical University Saitama Medical Center
Ehime University Hospital
Eiju General Hospital
Foundation for Detection of Early Gastric Carcinoma
Fuchu Hospital
Fujioka General Hospital
Fujisawa Shounandai Hospital
Fujita Health University Hospital
Fukui Prefectural Hospital
Fukui University Hospital
Fukui-ken Saiseikai Hospital
Fukuoka Dental College and Dental Hospital
Fukuoka Saiseikai General Hospital
Fukuoka University Chikushi Hospital
Fukuoka University Hospital
Fukuoka Wajiro Hospital
Fukushima Medical University Hospital
Fukuyama City Hospital
Fussa Hospital
Gifu Prefectural General Medical Center
Gifu University Hospital
Gunma Central General Hospital
Gunma Prefectural Cancer Center
Gunma University Hospital
Gunmaken Saiseikai Maebashi Hospital
Hachinohe City Hospital
Hakodate Goryokaku Hospital
Hakodate National Hospital
Hamamatsu University School of Medicine, University Hospital
Heartlife Hospital
Higashiosaka City Medical Center
Hino Memorial Hospital
Hino Municipal Hospital
Hiratsuka City Hospital
Hiratsuka Kyosai Hospital
Hirosaki University Hospital
Hiroshima City Asa Hospital
Hiroshima City Hiroshima Citizens Hospital
Hiroshima Red Cross Hospital & Atomic-bomb Survivors Hospital
Hiroshima University Hospital
Hitachi General Hospital
Hokkaido University Hospital
Hyogo Cancer Center
Hyogo Prefectural Nishinomiya Hospital
Ibaraki Prefectural Central Hospital
Iizuka Hospital
Imazu Surgical Clinic
Inazawa City Hospital
International University of Health and Welfare Hospital
International Goodwill Hospital
International University of Health and Welfare Mita Hospital
Isehara Kyodo Hospital
Ishikawa Prefectural Central Hospital
Iwate Medical University Hospital
Iwate Prefectural Central Hospital
Iwate Prefectural Chubu Hospital
Iwate Prefectural Isawa Hospital
Japanese Red Cross Fukui Hospital
Japanese Red Cross Ishinomaki Hospital
Japanese Red Cross Kyoto Daini Hospital
Japanese Red Cross Nagaoka Hospital
Japanese Red Cross Okayama Hospital
Japanese Red Cross Society Kyoto Daiichi Hospital
Japanese Red Cross Tottori Hospital
JCHO Kurume General Hospital
JCHO Kyushu Hospital
JCHO Miyazaki Konan Hospital
JCHO Osaka Hospital
JCHO Saitama Medical Center
JCHO Tokuyama Central Hospital
JCHO Yokohama Chuo Hospital
Jichi Medical University Hospital
Jichi Medical University Saitama Medical Center
Juntendo University Hospital
Juntendo University Shizuoka Hospital
Junwakai Memorial Hospital
Kagawa Prefectural Central Hospital
Kagawa Rosai Hospital
Kagawa University Hospital
Kagoshima Kenritsu Satsunan Hospital
Kagoshima University Hospital
Kaizuka City Hospital
Kameda General Hospital
Kanagawa Cancer Center
Kanazawa University Hospital
Kansai Medical University Hospital
Kansai Medical University Medical Center
Kansai Rosai Hospital
Kasamatsu Hospital
Kashiwa Kousei General Hospital
Kawakita General Hospital
Kawasaki Medical School Hospital
Kawasaki Medical School Kawasaki Hospital
Kawasaki Municipal Ida Hospital
Keio University Hospital
Keiyukai Sapporo Hospital
Kikuna Memorial Hospital
Kin-ikyo Chuo Hospital
Kinki Central Hospital
Kinki University Hospital
Kiryu Kosei General Hospital
Kishiwada City Hospital
Kitaakita Municipal Hospital
Kitakyushu Municipal Medical Center
Kitano Hospital
Kitasato Institute Hospital
Kitasato University Hospital
Kobe City Medical Center General Hospital
Kobe City Nishi-Kobe Medical Center
Kobe University Hospital
Kochi Health Sciences Center
Kochi University Hospital
Kokura Memorial Hospital
Kumagaya General Hospital
Kumamoto City Hospital
Kumamoto University Hospital
Kurashiki Central Hospital
Kurume University Hospital
Kusatsu General Hospital
Kyorin University Hospital
Kyoto University Hospital
Kyushu Central Hospital of the Mutual Aid Association of Public School Teachers
Kyushu Medical Center
Kyushu University Beppu Hospital
Kyushu University Hospital
Machida Municipal Hospital
Matsuda Hospital
Matsudo City Hospital
Matsushita Memorial Hospital
Matsuyama Red Cross Hospital
Mie University Hospital
Minamiosaka Hospital
Minamiyamato Hospital
Mino City Hospital
Mito Red Cross Hospital
Mitsui Memorial Hospital
Moriguchi Keijinkai Hospital
Murakami General Hospital
Musashino Red Cross Hospital
Nagahama City Hospital
Nagano Red Cross Hospital
Nagaoka Chuo General Hospital
Nagasaki University Hospital
Nagoya City East Medical Center
Nagoya City University Hospital
Nagoya City West Medical Center
Nagoya Daiichi Red Cross Hospital
Nagoya University Hospital
Nanpuh Hospital
Nara City Hospital
Nara Medical University Hospital
National Cancer Center Hospital
National Cancer Center Hospital East
National Defense Medical College Hospital
National Institute of Radiological Sciences Hospital
Nerima Hikarigaoka Hospital
NHO Beppu Medical Center
NHO Chiba Medical Center
NHO Chiba-East-Hospital
NHO Fukuoka-higashi Medical Center
NHO Himeji Medical Center
NHO Hokkaido Cancer Center
NHO Kanmon Medical Center
NHO Kure Medical Center
NHO Kyoto Medical Center
NHO Kyushu Cancer Center
NHO Matsumoto Medical Center
NHO Nagasaki Medical Center.
NHO Nagoya Medical Center
NHO Okayama Medical Center
NHO Osaka National Hospital
NHO Tokyo Medical Center
Nihonkai General Hospital
Niigata Cancer Center Hospital
Niigata City General Hospital
Niigata Prefectural Shibata Hospital
Niigata University Medical and Dental Hospital
Nikko Memorial Hospital
Nippon Medical School Chiba Hokusoh Hospital
Nippon Medical School Hospital
Nippon Medical School Musashi Kosugi Hospital
Nippon Medical School Tama Nagayama Hospital
Nishinomiya Municipal Central Hospital
NTT Medical Center Tokyo
NTT WEST Osaka Hospital
Numazu City Hospital
Obihiro Kousei Hospital
Ogachi Central Hospital
Ogaki Municipal Hospital
Ohta Nishinouchi Hospital
Oita Red Cross Hospital
Oita University Hospital
Okayama Saiseikai General Hospital
Okayama University Hospital
Omuta Tenryo Hospital
Osaka City General Hospital
Osaka City University Hospital
Osaka Ekisaikai Hospital
Osaka General Medical Center
Osaka International Cancer Institute
Osaka Medical College Hospital
Osaka Police Hospital
Osaka Red Cross Hospital
Osaka University Hospital
Otsu City Hospital
Rinku General Medical Center
Ryukyu University Hospital
Saga University Hospital
Saga-ken Medical Center Koseikan
Saiseikai Fukushima General Hospital
Saiseikai Kyoto Hospital
Saiseikai Utsunomiya Hospital
Saiseikai Yahata General Hospital
Saiseikai Yokohamashi Tobu Hospital
Saitama Cancer Center
Saitama City Hospital
Saitama Medical University Hospital
Saitama Medical University International Medical Center
Saitama Medical University Saitama Medical Center
Sakai City Medical Center
Saku Central Hospital
Sanin Rosai Hospital
Sendai City Hospital
Sendai Medical Center
Shiga General Hospital
Shiga University of Medical Science Hospital
Shikoku Cancer Center
Shimane University Hospital
Shimizu Welfare Hospital
Shinko Hospital
Shizuoka Cancer Center
Shizuoka City Shizuoka Hospital
Shizuoka General Hospital
Showa University Hospital
Showa University Koto-Toyosu Hospital
Sonoda Daiichi Hospital
Southern Tohoku General Hospital
St. Luke’s International Hospital
St. Marianna University School of Medical Hospital
Steel Memorial Hirohata Hospital
Sugita Genpaku Memorial Obama Municipal Hospital
Suita Municipal Hospital
Tachikawa Hospital
Takasago Municipal Hospital
Teikyo University Chiba Medical Center
Teikyo University Hospital
Teine Keijinkai Hospital
Tenri Hospital
The Jikei University Daisan Hospital
The Jikei University Hospital
Tochigi Cancer Center
Toho University Ohashi Medical Center
Toho University Omori Medical Center
Toho University Sakura Medical Center
Tohoku University Hospital
Tokai University Hachioji Hospital
Tokai University Hospital
Tokai University Tokyo Hospital
Tokushima Red Cross Hospital
Tokushima University Hospital
Tokyo Dental College Ichikawa General Hospital
Tokyo Medical and Dental University Hospital
Tokyo Medical University Hachioji Medical Center
Tokyo Medical University Hospital
Tokyo Medical University Ibaraki Medical Center
Tokyo Metropolitan Cancer and Infectious Diseases Center Komagome Hospital
Tokyo Metropolitan Tama Medical Center
Tokyo Saiseikai Central Hospital
Tokyo University Hospital
Tokyo Women’s Medical University Hospital
Tokyo Women’s Medical University Medical Center East
Tokyo Women’s Medical University Yachiyo Medical Center
Tonan Hospital
Toranomon Hospital
Toshima Hospital
Tottori Prefectural Central Hospital
Tottori University Hospital
Toyama Prefectural Central Hospital
Toyama University Hospital
Toyonaka Municipal Hospital
Tsuchiura Kyodo Hospital
Tsukuba University Hospital
Tsuruoka Municipal Shonai Hospital
University Hospital, Kyoto Prefectural University of Medicine
University of Miyazaki Hospital
Urasoe General Hospital
Wakayama Medical University Hospital
Yamagata Prefectural Central Hospital
Yamagata Prefectural Shinjo Hospital
Yamagata University Hospital
Yamaguchi University Hospital
Yamaguchi-ken Saiseikai Shimonoseki General Hospital
Yamanashi Prefectural Central Hospital
Yamanashi University Hospital
Yao Municipal Hospital
Yokohama City Municipal Hospital
Yokohama City University Medical Center
Yokohama Rosai Hospital
Yuri Kumiai General Hospital

(Total 316 institutions)

## Patient Background

Tables 1, 2, 3, 4, 5, 6, 7, 8.

**Table 1:** Age and gender

Age	Male	Female	Cases (%)
≤ 29	3	1	4 (0.0%)
30-39	16	14	30 (0.4%)
40-49	163	58	221 (2.8%)
50-59	1028	188	1216 (15.2%)
60-69	2814	453	3267 (40.8%)
70-79	2274	371	2645 (33.1%)
80-89	476	118	594 (7.4%)
90≤	18	8	26 (0.3%)
Total	6792	1211	8003

**Table 2:** Primary treatment

Treatments	Cases (%)
Surgery	4798 (60.0%)
Esophagectomy	4722 (59.0%)
Palliative surgery	76 (1.0%)
Chemotherapy/radiotherapy	1794 (22.4%)
Endoscopic treatment	1407 (17.6%)
Total	7999

**Table 3:** Tumor location

Location of tumor	Endoscopic treatment (%)	Surgery		Chemotherapy and/or radiotherapy (%)	Total (%)
		Esophagectomy (%)	Palliative surgery (%)		
Cervical	41 (2.9%)	152 (3.2%)	3 (3.9%)	174 (9.7%)	370 (4.6%)
Upper thoracic	122 (8.7%)	581 (12.3%)	18 (23.7%)	302 (16.8%)	1023 (12.8%)
Middle thoracic	819 (58.2%)	2151 (45.6%)	37 (48.7%)	825 (46.0%)	3832 (47.9%)
Lower thoracic	335 (23.8%)	1344 (28.5%)	14 (18.4%)	392 (21.9%)	2085 (26.1%)
EG	58 (4.1%)	356 (7.5%)	2 (2.6%)	39 (2.2%)	455 (5.7%)
E = G	14 (1.0%)	56 (1.2%)	1 (1.3%)	10 (0.6%)	81 (1.0%)
GE	4 (0.3%)	59 (1.2%)	1 (1.3%)	7 (0.4%)	71 (0.9%)
Unknown	14 (1.0%)	23 (0.5%)		45 (2.5%)	82 (1.0%)
Total	1407	4722	76	1794	7999

*E* esophageal, *G* gastric

**Table 4:** Histologic types of biopsy specimens

Histologic types	Cases (%)
Squamous cell carcinoma	7162 (89.5%)
Squamous cell carcinoma	5130 (64.1%)
Well differentiated	440 (5.5%)
Moderately differentiated	1177 (14.7%)
Poorly differentiated	415 (5.2%)
Adenocarcinoma	358 (4.5%)
Barrett’s adenocarcinoma	119 (1.5%)
Adenosquamous carcinoma	15 (0.2%)
Mucoepidermoid carcinoma	6 (0.1%)
Basaloid carcinoma	41 (0.5%)
Neuroendocrine cell tumor	24 (0.3%)
Undifferentiated carcinoma	12 (0.2%)
Sarcoma	79 (1.0%)
Malignant melanoma	1 (0.0%)
Carcinosarcoma	19 (0.2%)
GIST	14 (0.2%)
Other tumors	2 (0.0%)
Unknown	147 (1.8%)
Total	7999

**Table 5:** Depth of tumor invasion, cT (UICC TNM 7th)

cT	Cases (%)
cTX	100 (1.3%)
cT0	14 (0.2%)
cTis	216 (2.7%)
cT1a	1264 (15.8%)
cT1b	1575 (19.7%)
cT2	1004 (12.6%)
cT3	2843 (35.5%)
cT4a	395 (4.9%)
cT4b	538 (6.7%)
Unknown	50 (0.6%)
Total	7999

**Table 6:** Lymph node metastasis, cN (UICC TNM 7th)

cN	Cases (%)
cNX	170 (2.1%)
cN0	3796 (47.5%)
cN1	2020 (25.3%)
cN2	1335 (16.7%)
cN3	519 (6.5%)
Unknown	159 (2.0%)
Total	7999

**Table 7:** Distant metastasis, cM (UICC TNM 7th)

cM	Cases (%)
cM0	7214 (90.2%)
cM1	740 (9.3%)
Unknown	45 (0.6%)
Total	7999

**Table 8:** Clinical stage (UICC TNM 7th)

Clinical Stage	Endoscopic treatment(%)	Surgery		Chemotherapy and/or radiotherapy (%)	Total (%)
		Esophagectomy (%)	Palliative surgery (%)		
Stage 0	254 (18.1%)	104 (2.2%)	2 (2.6%)	54 (3.0%)	414 (5.2%)
Stage IA	981 (69.7%)	1117 (23.7%)	1 (1.3%)	201 (11.2%)	2300 (28.8%)
Stage IB	6 (0.4%)	393 (8.3%)		50 (2.8%)	449 (5.6%)
Stage IIA	5 (0.4%)	499 (10.6%)	6 (7.9%)	79 (4.4%)	589 (7.4%)
Stage IIB	3 (0.2%)	458 (9.7%)		88 (4.9%)	549 (6.9%)
Stage IIIA	9 (0.6%)	951 (20.1%)	14 (18.4%)	210 (11.7%)	1184 (14.8%)
Stage IIIB	10 (0.7%)	510 (10.8%)	6 (7.9%)	117 (6.5%)	643 (8.0%)
Stage IIIC	20 (1.4%)	384 (8.1%)	26 (34.2%)	418 (23.3%)	848 (10.6%)
Stage IV	36 (2.6%)	183 (3.9%)	16 (21.1%)	505 (28.1%)	740 (9.3%)
Unknown	83 (5.9%)	123 (2.6%)	5 (6.6%)	72 (4.0%)	283 (3.5%)
Total	1407	4722	76	1794	7999

### II. Results of endoscopically treated patients in 2012

Tables 9, 10, 11 and Figs. 1, 2, 3.

**Table 9:** Details of endoscopic treatment for curative intent

Treatment details	Cases (%)
EMR	185 (14.2%)
EMR + YAG laser	6 (0.5%)
EMR + MCT/RFA	1 (0.1%)
ESD	1064 (81.9%)
ESD + EMR	2 (0.2%)
ESD + PDT	8 (0.6%)
ESD + YAG laser	8 (0.6%)
PDT	3 (0.2%)
YAG laser	22 (1.7%)
Total	1299

*EMR* endoscopic mucosal resection, *PDT* photodynamic therapy, *YAG* yttrium aluminum garnet, *MCT* microwave coagulation therapy, *ESD* endoscopic submucosal dissection

**Table 10:** Complications of EMR/ESD

Complications of EMR/ESD	Cases (%)
None	1206 (94.7%)
Perforation	20 (1.6%)
Bleeding	1 (0.1%)
Stenosis	35 (2.7%)
Others	5 (0.4%)
Unknown	7 (0.5%)
Total	1274

**Table 11:** Pathological depth of tumor invasion of EMR/ESD specimens

Pathological depth of tumor invasion	Cases (%)
pTX	6 (0.5%)
pT0	10 (0.8%)
pTis	234 (18.4%)
pT1a	873 (68.5%)
pT1b	140 (11.0%)
pT2	2 (0.2%)
Unknown	9 (0.7%)
Total	1274

### III. Results in patients treated with chemotherapy and/or radiotherapy in 2012

Tables 12, 13 and Figs. 4, 5, 6.

**Table 12:** Dose of irradiation (non-surgically treated cases)

Dose of irradiation (Gy)	Definitive		Palliative (%)	Recurrence (%)	Others (%)	Unknown (%)	Total (%)
	Radiation alone (%)	Chemoradiotherapy (%)					
− 29	7 (3.2%)	14 (1.5%)	14 (4.5%)		1 (3.3%)		36 (2.4%)
30–39	3 (1.4%)	13 (1.4%)	46 (14.7%)	1 (20.0%)	5 (16.7%)	1 (2.5%)	69 (4.6%)
40–49	4 (1.9%)	36 (4.0%)	63 (20.1%)	1 (20.0%)	7 (23.3%)	1 (2.5%)	112 (7.4%)
50–59	35 (16.2%)	192 (21.2%)	76 (24.3%)		8 (26.7%)	10 (25.0%)	321 (21.3%)
60–69	127 (58.8%)	546 (60.4%)	103 (32.9%)	3 (60.0%)	7 (23.3%)	27 (67.5%)	813 (53.9%)
70-	7 (3.2%)	30 (3.3%)	4 (1.3%)		(0.0%)		41 (2.2%)
Unknown	33 (15.3%)	73 (8.1%)	7 (2.2%)		2 (6.7%)	1 (2.5%)	116 (7.7%)
Total	216	904	313	5	30	40	1508
Median (min–max)	60.0 (2.0–139.0)	60.0 (8.0–104.4)	50.0 (2.0–126.0)	60.0 (30.0–63.0)	50.0 (4.5–63.0)	60.0 (30.0–66.0)	60.0 (2.0–139.0)

**Table 13:** Dose of irradiation (surgically treated cases)

Dose of irradiation (Gy)	Preoperative irradiation (%)	Postoperative irradiation (%)
− 29	3 (1.0%)	1 (1.8%)
30–39	86 (28.6%)	3 (5.4%)
40–49	166 (55.1%)	9 (16.1%)
50–59	10 (3.3%)	17 (30.4%)
60–69	23 (7.6%)	23 (41.1%)
70–		
Unknown	13 (4.3%)	3 (5.4%)
Total	301	56
Median (min–max)	40.0 (3.0–66.6)	50.5 (16.0–66.0)

### IV. Results in patients who underwent esophagectomy in 2012

Tables 14,15,16,17,18,19,20,21,22,23,24,25,26,27, and Figs. 7, 8, 9, 10, 11, 12, 13, 14, 15.

**Table 14:** Treatment modalities of esophagectomy

Treatments	Cases (%)
Esophagectomy	1792 (38.0%)
Esophagectomy + endoscopic treatment	123 (2.6%)
Esophagectomy + chemoradiotherapy	806 (17.1%)
Concurrent chemoradiotherapy	557 (11.8%)
Other	249 (5.3%)
Esophagectomy + chemoradiotherapy + endoscopic treatment	24 (0.5%)
Esophagectomy + chemoradiotherapy + other treatment	1 (0.0%)
Esophagectomy + radiotherapy	67 (1.4%)
Preoperative	15 (0.3%)
Postoperative	23 (0.5%)
Recurrence	4 (0.1%)
Other	25 (0.5%)
Esophagectomy + chemotherapy	1886 (39.9%)
Preoperative	1473 (31.2%)
Postoperative	253 (5.4%)
Recurrence	52 (1.1%)
Other	108 (2.3%)
Esophagectomy + chemotherapy + endoscopic treatment	21 (0.4%)
Esophagectomy + chemotherapy + other treatment	1 (0.0%)
Esophagectomy + other treatment	1 (0.0%)
Total	4722

**Table 15:** Tumor location

Locations	Cases (%)
Cervical	152 (3.2%)
Upper thoracic	581 (12.3%)
Middle thoracic	2151 (45.6%)
Lower thoracic	1344 (28.5%)
*E* > *G*	356 (7.5%)
*E* = *G*	56 (1.2%)
*G* > *E*	59 (1.2%)
Unknown	23 (0.5%)
Total lesions	4722

**Table 16:** Approaches to tumor resection

Approaches	Cases (%)
Cervical approach	83 (1.8%)
Right thoracic	4070 (86.2%)
Left thoracic	81 (1.7%)
Left thoracoabdominal	80 (1.7%)
Abdominal	173 (3.7%)
Transhiatal thoracic esophagectomy	59 (1.2%)
Transhiatal lower esophagectomy	115 (2.4%)
Sternotomy	9 (0.2%)
Others	25 (0.5%)
Unknown	27 (0.6%)
Total	4722

Thoracic includes thoracotomy and thoracoscopic

Abdominal includes laparotomy and laparoscopic

**Table 17:** Video-assisted surgery

Video-assisted surgery	Cases (%)
None	2623 (55.5%)
Thoracoscopy	983 (20.8%)
Thoracoscopy + laparoscopy	712 (15.1%)
Thoracoscopy + laparoscopy + mediastinoscopy	3 (0.1%)
Thoracoscopy + laparoscopy + other	1 (0.0%)
Thoracoscopy + mediastinoscopy	3 (0.1%)
Thoracoscopy + other	2 (0.0%)
Laparoscopy	218 (4.6%)
Laparoscopy + mediastinoscopy	11 (0.2%)
Laparoscopy + mediastinoscopy + other	13 (0.3%)
Mediastinoscopy	41 (0.9%)
Others	107 (2.3%)
Unknown	5 (0.1%)
Total	4722

**Table 18:** Fields of lymph node dissection according to the location of the tumor

Field of lymphadenectomy	Cervical	Upper thoracic	Middle thoracic	Lower thoracic	*E* > *G*	*E* = *G*	*G* > *E*	Unknown	Total
None	19 (12.5%)	27 (4.6%)	81 (3.8%)	39 (2.9%)	17 (4.8%)	3 (5.4%)	1	1 (4.3%)	188 (4.0%)
C	40 (26.3%)	8 (1.4%)	8 (0.4%)	5 (0.4%)	(0.0%)				61 (1.3%)
C + UM	16 (10.5%)	3 (0.5%)	3 (0.1%)	2 (0.1%)		1 (1.8%)		1	26 (0.6%)
C + UM + MLM	11 (7.2%)	25 (4.3%)	43 (2.0%)	18 (1.3%)	1 (0.3%)				98 (2.1%)
C + UM + MLM + A	45 (29.6%)	369 (63.5%)	1103 (51.3%)	506 (37.6%)	39 (11.0%)	3 (5.4%)	1 (1.7%)	6 (26.1%)	2072 (43.9%)
C + UM + MLM + A + OT		1 (0.1%)							1 (0.0%)
C + UM + A	3 (2.0%)	5 (0.9%)	1 (0.0%)	2 (0.1%)					11 (0.2%)
C + MLM			1(0.0%)	1 (0.1%)					2 (0.0%)
C + MLM + A		4 (0.3%)	7 (0.3%)	5 (0.4%)	1 (0.3%)				17 (0.4%)
C + A	2 (1.3%)		4 (0.2%)	1 (0.1%)					7 (0.1%)
UM			5 (0.2%)	2 (0.1%)	3 (0.8%)				10 (0.2%)
UM + MLM	1 (0.7%)	11 (1.9%)	33 (1.5%)	14 (1.0%)	4 (1.1%)			2 (8.7%)	65 (1.4%)
UM + MLM + A	10 (6.6%)	110 (18.9%)	768 (35.7%)	595 (44.3%)	115 (32.3%)	11 (19.6%)	7 (11.9%)	8 (34.8%)	1624 (34.4%)
UM + MLM + A + OT			1 (0.1%)						1 (0.0%)
UM + A			7 (0.3%)	3 (0.2%)	1 (0.3%)				11 (0.2%)
MLM		1 (0.2%)	9 (0.4%)	7 (0.5%)	4 (1.1%)				21 (0.4%)
MLM + A		8 (1.4%)	45 (2.1%)	105 (7.8%)	129 (36.2%)	26 (46.4%)	27 (45.8%)	2 (8.7%)	342 (7.2%)
A	1 (0.7%)	3 (0.5%)	21 (1.0%)	29 (2.2%)	40 (11.2%)	9 (16.1%)	23 (39.0%)	1 (4.3%)	127 (2.7%)
A + OT				1 (0.1%)					1 (0.0%)
Unknown	4 (2.6%)	6 (1.0%)	11 (0.5%)	9 (0.7%)	2 (0.6%)	3 (5.4%)		2 (8.7%)	37 (0.8%)
Total	152	581	2151	1344	356	56			4722

*C* bilateral cervical nodes, *UM* upper mediastinal nodes, *MLM* middle-lower mediastinal nodes, *A* abdominal nodes

**Table 19:** Reconstruction route

Route	Cases (%)
None	66 (1.4%)
Subcutaneous	414 (8.8%)
Retrosternal	1799 (38.1%)
Posterior mediastinal	519 (11.0%)
Intrathoracic	1794 (38.0%)
Cervical	46 (1.0%)
Others	49 (1.0%)
Unknown	35 (0.7%)
Total	4722

**Table 20:** Organs used for reconstruction

Organs used for reconstruction	Cases (%)
None	62 (1.3%)
Whole stomach	49 (1.0%)
Gastric tube	4057 (85.0%)
Jejunum	286 (6.0%)
Free jejunum	94 (2.0%)
Colon	157 (3.3%)
Free colon	12 (0.3%)
Others	24 (0.5%)
Unknown	33 (0.7%)
Total organs	4774
Total cases	4722

**Table 21:** Histological classification

Histological classification	Cases (%)
Squamous cell carcinoma	3990 (84.5%)
Squamous cell carcinoma	973 (20.6%)
Well differentiated	713 (15.1%)
Moderately differentiated	1788 (37.9%)
Poorly differentiated	516 (10.9%)
Adenocarcinoma	239 (5.1%)
Barrett’s adenocarcinoma	109 (2.3%)
Adenosquamous carcinoma	30 (0.6%)
Mucoepidermoid carcinoma	5 (0.1%)
Adenoid cystic carcinoma	3 (0.1%)
Basaloid carcinoma	81 (1.7%)
Neuroendocrine cell tumor	21 (0.4%)
Undifferentiated carcinoma	12 (0.3%)
Other carcinoma	6 (0.1%)
Carcinosarcoma	36 (0.8%)
Malignant melanoma	11 (0.2%)
GIST	1 (0.0%)
Other	46 (1.0%)
Unknown	132 (2.8%)
Total	4722

**Table 22:** Pathological depth of tumor invasion, pT (JES 10th)

Pathological depth of tumor invasion	Cases (%)
pTX	71 (1.5%)
pT0	156 (3.3%)
pTis	33 (0.7%)
pT1a	503 (10.7%)
pT1b	1256 (26.6%)
pT2	570 (12.1%)
pT3	1824 (38.6%)
pT4	20 (0.4%)
pT4a	147 (3.1%)
pT4b	103 (2.2%)
Unknown	39 (0.8%)
Total	4722

**Table 23:** Pathological grading of lymph node metastasis, pN (JES 10th)

Lymph node metastasis	Cases (%)
pN0	2237 (47.4%)
pN1	626 (13.3%)
pN2	1172 (24.8%)
pN3	393 (8.3%)
pN4	226 (4.8%)
Unknown	68 (1.4%)
Total	4722

**Table 24:** Pathological findings of lymph node metastasis, pN (UICC 7th)

Lymph node metastasis	Cases (%)
pN0	2164 (45.8%)
pN1 (1-2)	1265 (26.8%)
pN2 (3-6)	790 (16.7%)
pN3 (7-)	392 (8.3%)
Unknown	111 (2.4%)
Total	4722

Regional lymph nodes are different in JES 10th and UICC 7th

Data for Tables 23 and 24 were analyzed from different variables in the registration application (Tables 25, 26, 27)

**Table 25:** Pathological findings of distant organ metastasis, pM (JES 10th)

Distant metastasis	Cases (%)
pMX	172 (3.6%)
pM0	4490 (95.1%)
pM1	60 (1.3%)
Total	4722

**Table 26:** Residual tumor

Residual tumor	Cases (%)
RX	156 (3.3%)
R0	4160 (88.1%)
R1	216 (4.6%)
R2	190 (4.0%)
Total	472

**Table 27:** Causes of death

Cause of death	Cases (%)
Death due to recurrence	1383 (71.5%)
Death due to other cancer	101 (5.2%)
Death due to other disease (with recurrence)	46 (2.4%)
Death due to other disease (without recurrence)	244 (12.6%)
Death due to other disease (recurrence unknown)	19 (1.0%)
Operative death^a^	25 (1.3%)
Postoperative hospital death^b^	40 (2.1%)
Unknown	77 (4.0%)
Total of death cases	1935

Operative mortality rate: 0.52%

Hospital mortality rate: 2.35%

^a^Operative death means death within 30 days after operation in or out of hospital

^b^Hospital death is defined as death during the same hospitalization, regardless of department at time of death